# Immunogene therapy with fusogenic nanoparticles modulates macrophage response to *Staphylococcus aureus*

**DOI:** 10.1038/s41467-018-04390-7

**Published:** 2018-05-17

**Authors:** Byungji Kim, Hong-Bo Pang, Jinyoung Kang, Ji-Ho Park, Erkki Ruoslahti, Michael J. Sailor

**Affiliations:** 1 Materials Science and Engineering Program, University of California, San Diego, 9500 Gilman Drive, La Jolla, California 92093 USA; 2 Cancer Research Center, Sanford Burnham Prebys Medical Discovery Institute, La Jolla, California 92037 USA; 3 Department of Pharmaceutics, University of Minnesota, Minneapolis, Minnesota 55455 USA; 4 Department of Nanoengineering, University of California, San Diego, 9500 Gilman Drive, La Jolla, California 92093 USA; 5 Department of Bio and Brain Engineering, Korea Advanced Institute of Science and Technology (KAIST), Daejeon, 34141 Republic of Korea; 6 Center for Nanomedicine and Department of Cell, Molecular and Developmental Biology, University of California, Santa Barbara, Santa Barbara, California 93106-9610 USA; 7 Department of Chemistry and Biochemistry, University of California, San Diego, 9500 Gilman Drive, La Jolla, California 92093 USA

## Abstract

The incidence of adverse effects and pathogen resistance encountered with small molecule antibiotics is increasing. As such, there is mounting focus on immunogene therapy to augment the immune system’s response to infection and accelerate healing. A major obstacle to in vivo gene delivery is that the primary uptake pathway, cellular endocytosis, results in extracellular excretion and lysosomal degradation of genetic material. Here we show a nanosystem that bypasses endocytosis and achieves potent gene knockdown efficacy. Porous silicon nanoparticles containing an outer sheath of homing peptides and fusogenic liposome selectively target macrophages and directly introduce an oligonucleotide payload into the cytosol. Highly effective knockdown of the proinflammatory macrophage marker IRF5 enhances the clearance capability of macrophages and improves survival in a mouse model of *Staphyloccocus aureus* pneumonia.

## Abstract (web summary)

In the context of increasing bacterial antibiotic-resistance, gene therapy that targets the immune system to clear infection is a major goal. Here the authors show a silicon based nanosystem that modulates the macrophage response in an in vivo model of Staphylococcal pneumonia.

## Introduction

Deep-tissue *Staphyloccocus aureus* infection is a major therapeutic challenge. *S. aureus* is a Gram-positive bacterium that predominantly infects the skin and the respiratory system causing pneumonia; local infections can become systemic in the most serious form of Staphylococcal disease, sepsis^1^. At high levels of bacterial burden in the lungs, Staphyloccocal pneumonia becomes fatal due to two major factors: (1) pathogenic activity by *S. aureus* and (2) prolonged inflammation caused by the body’s immune system. The acute inflammatory response at the site of an infection involves the secretion of cytokines by alveolar macrophages, recruiting polymorphonuclear neutrophils (PMN) and monocytes from circulation that differentiate into macrophages^2^. Alveolar inflammation causes extensive bleeding and exudation that slow down vascular flow and impede breathing^2,3^, and prolonged excretion of inflammatory cytokines reduces the chances of recovery^3^. Although the immediate inflammatory response to Staphylococcal pneumonia is necessary for rapid elimination of the threat, it must be balanced with inflammation suppression and tissue repair to maintain lung homeostasis^4^.

Owing to toxic adverse effects of small molecule antibiotics such as vancomycin^5^ and the emergence of strains resistant to these therapeutics^2^ therapies are needed to activate the immune system to treat bacterial infections^6–9^. Macrophages are a potential target for such therapies owing to their polar functions as inflammatory, immune stimulatory phagocytes M1 macrophages, or as anti-inflammatory phagocytic M2 macrophages associated with bacterial phagocytosis and tissue repair functions^10–15^. M1 macrophages are marked by the *Irf5* gene, which upregulates tumor necrosis factor (TNF), interleukin (IL)-1, IL-6, IL-15, IL-18, and IL-23, and downregulates anti-inflammatory cytokines such as IL-10^10,12–15^. Knockdown of *Irf5* in the early stages of Staphylococcal pneumonia can curtail prolonged inflammation by preventing the excretion of inflammatory cytokines, allowing the immune system to clear bacteria and repair tissue^10,15,16^.

Despite much effort, in vivo knockdown of genes has still not been of great success. Naked RNA has a short half-life in vivo; thus, various types of nanoparticle (NP) delivery vehicles have been used to protect the oligonucleotide and deliver it intracellularly^17–19^. The most common means of delivery is with lipid NPs^20^, which are readily endocytosed by the cell, leading to extracellular excretion of 70% of the small interfering RNA (siRNA) payload, with the remaining siRNA undergoing lysosomal degradation. Typically, only 1–2% of administered siRNA escapes early endosomal uptake to potentially undergo RNA interference (RNAi)^21–23^. In order to increase the quantity of RNA delivered, polymeric and related hybrid NPs have been engineered with cationic polyethylenimine (PEI) components. Although it increases the carrying capacity of the NPs, PEI is also cytotoxic^24,25^. Some lipid constituents, such as dioleoylphosphatidylethanolamine or 1,2-dioleoyl-3-trimethylammonium-propane (DOTAP), impart a fusogenic nature to liposomes that enables them to fuse with the cellular membrane, mitigating toxicity, and enhancing cellular delivery of genes^26–29^. With some PEGylated lipid compositions, fusogenic liposomes have been shown to bypass endocytosis altogether, much like the endogenous soluble N-ethylmaleimide-sensitive factor attachment protein receptor (SNARE)-mediated vesicular uptake mechanism^30,31^.

Although cellular penetration is important, gene therapeutics must also reach the appropriate cell to be effective^18,19,32–34^. Here we present a solution to these problems that uses NPs containing a targeting peptide specific for activated macrophages and a fusogenic liposomal coating (F-pSi). Membrane fusion enables direct release of hydrophilic payloads from the core of NP directly into the cell cytoplasm, the transfer of hydrophobic molecules from the liposomal bilayer to the cell membrane bilayer, and the transfer of moieties conjugated on the outer surface of the lipid coat (including antibodies, small molecules, and peptides) to the cell membrane. By avoiding endocytosis entirely, the fusogenic coating increases the probability that siRNAs will reach the perinuclear region to undergo RNAi. In addition, in place of conventional peptide-based and polymeric NPs (such as protamine, poly-l-lysine, and PEI), we use porous silicon NPs (pSiNPs), which have been shown to be an effective gene delivery vehicle^35^. The pSiNPs are prepared with a calcium silicate trapping chemistry^36^ that can load and protect high quantities of siRNA without the use of cytotoxic polymer stabilizers. We use siRNA against *Irf5* to inhibit the inflammatory phenotype of macrophages and favor phagocytotic function. These macrophage-targeting F-pSi hybrid NPs have high siRNA knockdown efficiency in vitro and provide strong therapeutic efficacy against a *S. aureus* infection in mice, affording full recovery from a lethal dose. This study is the first successful in vivo demonstration of gene silencing for immunotherapy of deep-tissue infection, with implications for the treatment of antibiotic-resistant bacterial infections.

## Results

### Synthesis of fusogenic lipid-coated pSiNPs

**Fig. 1:**
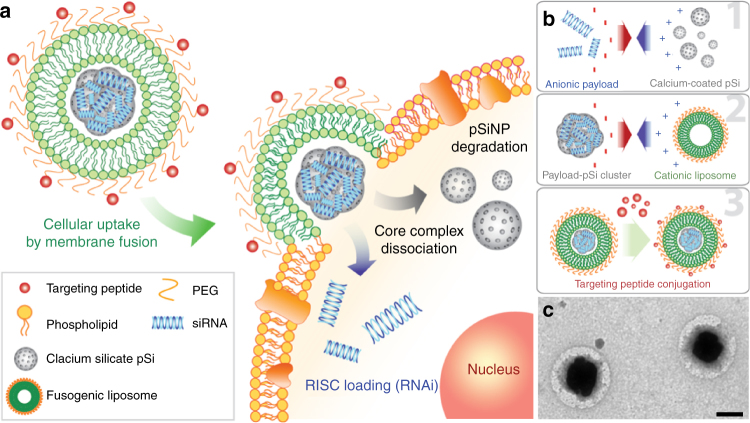
Fusogenic pSi nanoparticle system (F-pSi). **a** Schematic showing mode of action of the fusogenic pSiNP. **b** Schematic showing nanoparticle synthesis, including (1) siRNA loading into the porous silicon nanoparticles and sealing by precipitation of calcium silicate; (2) coating of the nanoparticle clusters with cationic liposome; and (3) conjugation of targeting peptides to the liposomal exterior. **c** TEM image of final F-pSi constructs, showing cloudy liposomal coatings around dark and dense porous silicon-based cores. Imaged using JEOL 1200 EX TEM. Negative staining by 2% phosphotungstic acid. Scale bar indicates 200 nm

The siRNA carrier consisted of pSiNPs, prepared by electrochemical etch of single-crystal silicon wafers, and ultrasonic fracture of the resulting porous layers into NPs. siRNA and hydrophilic fluorescent dye payload were loaded into the porous NPs by subjecting the payload and the pSiNPs to ultrasound in an aqueous solution of calcium chloride^36^. Fusogenic liposomes were synthesized using the established film hydration method^30,31^ and were coated around the payload-loaded pSiNPs by co-extrusion through 200 nm polycarbonate membranes (Fig. 1b). The fusogenic (F) feature of the liposomes is derived from a controlled ratio of structural, cationic, and PEGylated lipid components^30,31^ (Supplementary Table 1). Control non-fusogenic (NF) NPs were prepared using the structural and PEGylated lipids, but without the cationic component—yielding a more conventional liposome. Dynamic light scattering (DLS) and microscopic data on the F-pSiNPs confirmed an average hydrodynamic diameter of ~ 190 nm with a distribution range of 100–400 nm, with a cationic surface charge of ~ 10 mV (Supplementary Table 2). The F-pSi formulations were physically stable in deionized water for up to 28 days at 4 °C (Supplementary Fig. 1). The loading efficiency of the siRNA payload was ~ 25 wt%, substantially larger than the 1–14 wt% achieved with other reported oligonucleotide-loaded nanoplatforms, such as lipid-based NPs and mesoporous Si-polymer hybrid systems (Supplementary Table 3). Notably, particles with sizes comparable to those used in the present study (200 nm) have displayed oligonucleotide loadings of < 5 wt%.

### Intracellular delivery of fusogenic pSiNPs in vitro

**Fig. 2:**
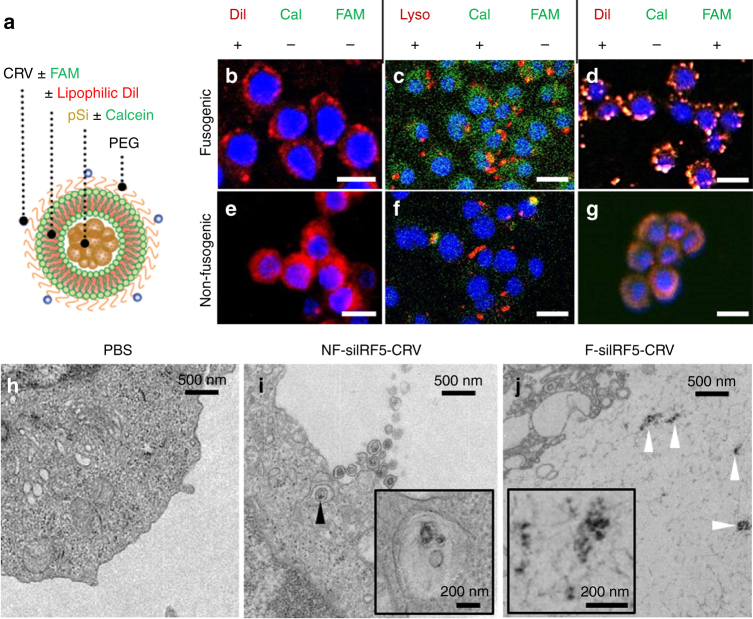
Fusion and intracellular delivery of Fusogenic particles in vitro. **a** Particle schematic. Calcein, anionic calcein fluorescent dye; CRV, macrophage-targeting peptide; DiI, the hydrophobic carbocyanine membrane stain; FAM, fluorescein label attached to targeting peptide; PEG, polyethylene glycol; pSi, porous Si nanoparticles. **b**–**g** Confocal microscope images of J771A.1 murine macrophage cells; **b** after 10 min incubation with DiI-loaded F-pSi nanoparticles; **c** after 1 h incubation with Lysotracker Red and 10 min incubation with calcein-loaded F-pSi nanoparticles; **d** after 5 min incubation with CRV-FAM-conjugated, DiI-loaded F-pSi nanoparticles; **e** after 10 min incubation with DiI-loaded NF-pSi nanoparticles; **f** after 1 h incubation with Lysotracker Red and 10 min incubation with calcein-loaded NF-pSi nanoparticles; **g** after 5 min incubation with CRV-FAM-conjugated, DiI-loaded NF-pSi nanoparticles. Blue is DAPI nuclear stain. **h**–**j** Transmission electron microscope (TEM) images of Raw 264.7 murine macrophage cells after 10 min incubation with nanoparticles. **h** Cells treated with PBS (phosphate-buffered saline) control show no signs of particles; **i** cells treated with nanoparticles containing a non-fusogenic lipid coating, siRNA against transcription factor *Irf5*, and the macrophage-targeting peptide (NF-siIRF5-CRV) display evidence of pinocytotic uptake (arrowhead). Inset shows particles localized in vesicles (endosome/lysosome); **j** cells treated with nanoparticles containing fusogenic lipid coating, siRNA against transcription factor IRF5, and the macrophage-targeting peptide (F-siIRF5-CRV) become localized in the cell cytoplasm. Scale bar represents 20 µm

NPs were loaded with the hydrophilic dye calcein in the pSi core or with the lipophilic dye 1,1’-dioctadecyl-3,3,3’,3’-tetramethylindocarbocyanine perchlorate (DiI) in the lipid leaflets to evaluate differences in intracellular localization and to infer uptake pathway. Calcein was chosen as the model-siRNA payload (Fig. 2a), because it shares two key characteristics with oligonucleotides: (i) it is anionic and (ii) it is membrane impermeable. The lipophilic DiI was chosen to track fusogenic uptake, because the hydrophobic molecule localizes in lipid bilayers (Fig. 2a)^37^. In the event of fusogenic uptake, DiI would be expected to diffuse from the liposomal bilayer of the NP into the plasma membrane, whereas NF uptake would result in endocytosis and localization in the cytoplasm.

Confocal microscopy revealed that the F-pSi formulation fused with the plasma membrane of cultured J774A.1 murine macrophages and delivered the payload into the cytoplasm. The F-pSi formulation that contained DiI in the liposomal coating transferred this lipophilic dye to the cell membrane (Fig. 2b), confirming fusion, whereas the F-pSi formulation that contained calcein in the NP interior dispersed this hydrophilic dye throughout the cytoplasm (Fig. 2c). Control experiments performed using NF NPs resulted in an overall lower uptake and the DiI signal from the NF formulation were found to be concentrated within the cytoplasm (Fig. 2e). The fact that these NPs were associated with intracellular endosomes/lysosomes was confirmed using LysoTracker Red stain (Figs. 2c, 2f). By contrast, fusogenic NPs showed dispersed calcein signals that did not co-localize with lysosomal compartments. The F-pSiNPs attained an average Pearson’s correlation coefficient (PCC) = 0.04 ± 0.03, whereas the NF-pSiNPs attained an average PCC = 0.59 ± 0.18. The two values are significantly different by *T*-test (*p* = 2 × 10^−6^), validating the lysosomal compartmentalization of the NF-pSi formulation.

The ability of the nanoparticles to be selectively targeted to macrophages was tested using a peptide selected in a phage library screen^38,39^ for cultured J774A.1 murine macrophages (Supplementary Fig 2a). This peptide, denoted as CRV, is a nine-amino acid peptide (sequence CRVLRSGSC) made cyclic by a disulfide bond between the side chains of the two cysteine residues. CRV labeled with a 5-FAM dye (FAM-CRV) showed higher binding to J774A.1 and Raw 264.7 macrophages relative to control peptides (Supplementary Fig. 2b). Biotin-labeled CRV was able to significantly reduce the binding of FAM-CRV to the macrophages (Supplementary Fig. 2c). All these results suggest that CRV peptide specifically binds to macrophages.

To permit coupling to the NPs, the peptide was modified by adding a third cysteine through a 6-aminohexanoic acid linker and labeled with a 5-FAM dye in order to allow tracking by fluorescence. The 3-cysteine peptide was attached to the polyethylene glycol (PEG) head of a minor fraction of the lipids via maleimide coupling chemistry. A fusogenic formulation containing CRV, the DiI membrane dye, and pSiNPs (F-DiI-CRV) showed strong colocalization of the DiI and FAM signals at specific points on the cell membrane (Fig. 2d), suggesting that CRV anchors the particles to its specific macrophage membrane receptors to allow localized fusion. The data are more consistent with a membrane fusion mechanism rather than endocytosis; substantial endocytosis would be expected to lead to increased DiI signals from the cell cytoplasm. We note that CRVs appeared to expedite the fusion process, as only half the incubation time was needed to achieve a comparable level of fusion. In contrast, the NF-DiI-CRV construct (Fig. 2g) displayed a cellular distribution similar to NF-DiI-mPEG, a NF formulation that contained mPEG (methoxy PEG) in place of the targeting peptide-conjugated PEG (Fig. 2e). The CRV-FAM and DiI signals colocalized in clusters within the cytoplasm, indicative of endosomal and lysosomal compartmentalization. These results indicate the targeting peptide is permissive for either cell entry pathway.

Transmission electron microscope (TEM) results (Fig. 2h–j) verify the different uptake pathways between fusogenic and NF formulations. Although the targeted NF particles demonstrated macropinocytosis, and were found to be localized in intracellular vesicles (Fig. 2i), the pSi cores of the targeted fusogenic particles were found degrading in the cell cytoplasm (Fig. 2j).

### Biosafety and in vitro knockdown using fusogenic pSiNPs

**Fig. 3:**
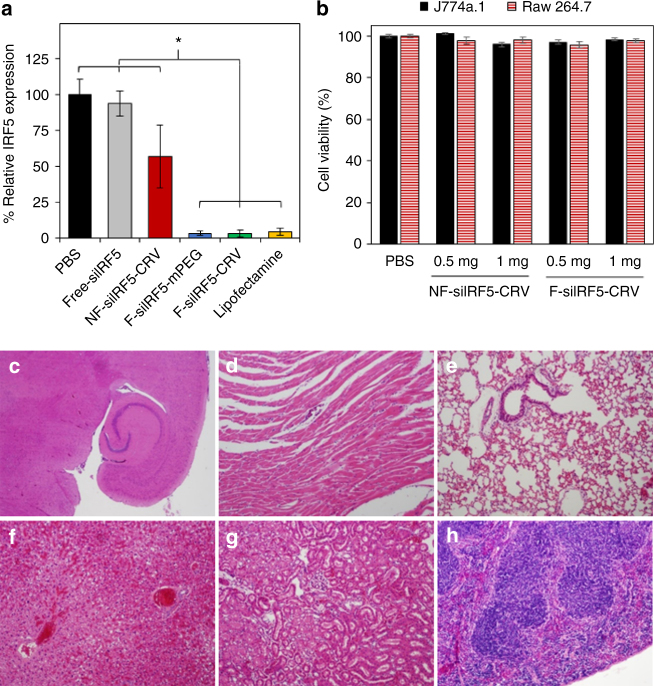
Gene knockdown in vitro and in vivo cytotoxicity of fusogenic porous Si nanoparticle constructs. **a** siRNA knockdown results (via qRT-PCR) from Raw 264.7 macrophage cells incubated with nanoparticles for 24 h. Error bars indicate SD (*n* = 6). The fusogenic formulations show substantial knockdown, comparable to standard lipofectamine transfection agent. No significant difference in knockdown efficiency is observed between the two fusogenic formulations (F-siIRF5-mPEG, F-siIRF5-CRV) and lipofectamine. *Significant difference (one-way ANOVA with Tukey’s HSD post hoc test, *p*-level < 0.05, *F* (5, 30) = 28, *p* = 6.9 × 10^−10^). **b** Viability of J774a.1 and Raw 264.7 macrophage cells after 1 h incubation with NF-siIRF5-CRV and F-siIRF5-CRV nanoparticle constructs, containing 0.5 and 1 mg total mass of lipid as indicated. Error bar indicates SD (*n* = 6). ANOVA test found no statistical significance at *p* < 0.01; **c**–**h** H&E staining of major organs after 24 h circulation of F-siIRF5-CRV via tail vein injection (23.2 µmol/kg lipid, 24 µg/kg siRNA, 0.5 mg/kg pSi) in healthy Balb/C mice; **c** brain; **d** heart; **e** lung; **f** liver; **g** kidney; and **h** spleen

To test the hypothesis that the fusogenic pathway for uptake and direct cytoplasmic release of siRNA attains higher knockdown efficiency than the endosomal uptake route, we delivered siRNA encoding the *Irf5* gene to cultured Raw 264.7 macrophage cells and gene expression was analyzed by quantitative real-time PCR (qRT-PCR) (Fig. 3a). Fusogenic NP formulations (F-siIRF5) delivering 200 nM siRNA demonstrated high knockdown efficiencies with or without an added CRV macrophage-targeting peptide (96.4% and 96.6%, respectively). Knockdown was comparable to that of the standard transfection agent, Lipofectamine (95.5%). The NF formulation (NF-siIRF5-CRV) demonstrated only 42.9% knockdown with high SD. The relatively high variance in this control is attributed to occasional incidences of endosomal escape^22^. Administration of free si*Irf5* achieved only 6.3% knockdown under similar conditions, statistically similar to the phosphate-buffered saline (PBS) control. The presence of CRV peptides on the particle surface had no adverse effect on knockdown ability. These experiments established that the fusogenic coatings enable higher knockdown efficiency than a conventional liposomal coating, and that active targeting of the fusogenic formulations does not enhance gene silencing in the isolated cellular environment of the in vitro experiment, likely because the long incubation period allowed the entry of non-targeted NP to catch up with the targeted NPs.

Assays of cell cytotoxicity and histological evaluations of major organs were performed to assess biosafety of the fusogenic NPs and their siRNA payload. The primary concern with the fusogenic system is the insertion of the cationic lipid constituents into cell membranes, disrupting membrane charge homeostasis in the cells. Two macrophage cell lines, J774a.1 and Raw 264.7, were incubated for 1 h with NP formulations containing 0.5 and 1 mg total lipid mass. Calcein AM and ethidium homodimer-1 (EthD-1) assays indicated > 95% cell viability compared with the PBS-treated control for either fusogenic or NF CRV-targeted NP constructs (Fig. 3b). To test in vivo biosafety, healthy Balb/C mice were intravenously injected with the fusogenic, CRV-targeted NP construct (F-siIRF5-CRV) at doses corresponding to 23.2 µmol/kg lipid, corresponding to 24 µg/kg siRNA. After 24 h circulation, the major organs were harvested and sectioned for hematoxylin and eosin (H&E) histopathological evaluation (Fig. 3c–h). All major organs were found to be normal, although the liver showed minor incidental findings also seen in control mice.

### Biodistribution and targeting of fusogenic pSiNPs

**Fig. 4:**
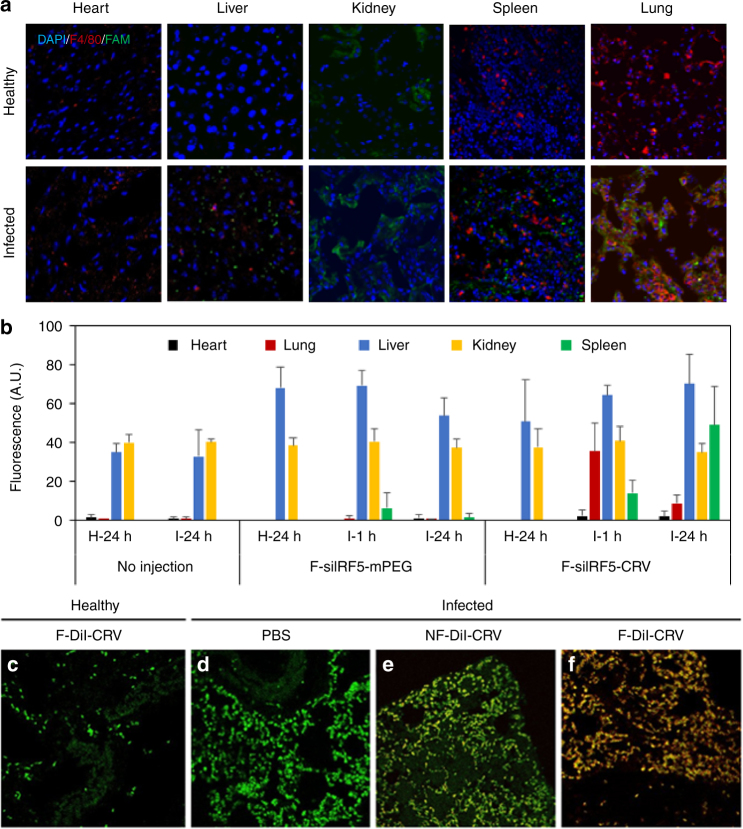
Targeting peptide and fusogenic uptake enhances homing to infected lungs and macrophages. **a** Immunofluorescent sections of major organs of healthy and infected Balb/C mice injected with FAM-CRV.peptide (green). Blue indicates cell nuclei stained with DAPI, red indicates macrophages marked by F4/80 antibody stain. **b** Quantified fluorescence signals (IVIS 200) from organs of healthy and infected Balb/C mice injected with fusogenic nanoparticles containing DiI membrane stain and si*Irf5* payload, with either a non-targeting (F-siIRF5-mPEG) or the CRV targeting group (F-siIRF5-CRV) at doses of 23.2 µmol/kg lipid, 24 µg/kg siRNA, 0.5 mg/kg pSi. “H-24” indicates healthy Balb/C organs "collected 24 h post treatment; “I-1” indicates Balb/C organs of infected harvested 1 h post; treatment; and “I-24” indicates infected Balb/C organs 24 h post-" treatment. Error bars indicate SD. Data are representative of *n* = 3, quantified by ImageJ analyses. **c**–**f** Confocal microscope images of DiI-loaded fusogenic and non-fusogenic nanoparticles homed to infected lung with pendant CRV targeting peptide. These nanoparticles contained no si*Irf5* payload; green indicates macrophages (FITC-tagged rat anti-mouse F4/80 stain), red indicates lipophilic DiI from nanoparticles; **c** lung of healthy mouse injected with F-DiI-CRV; **d** lung of infected mouse injected with PBS control; **e** lung of infected mouse injected with NF-DiI-CRV; **f** lung of infected mouse injected with F-DiI-CRV. The data show DiI from the fusogenic, CRV-targeted nanoparticles is strongly co-localized with macrophages

We established a *S. aureus* pneumonia animal model involving intratracheal injection of bacteria at a dose that was lethal to untreated mice within 2 days. First, we tested the homing ability of the CRV macrophage-targeting peptide in this model, using the “free” peptide not bound to any NP, but conjugated to a fluorescein (5-FAM) dye to allow tracking. Infected and healthy control mice (*n* = 3 each) were intravenously injected with FAM-CRV 24 h post infection. Confocal immunofluorescence microscopy of organs collected from the healthy animals showed signs of renal clearance but no selective homing to any of the macrophage-heavy organs (Fig. 4a). However, the infected animals demonstrated clear evidence of selective homing, with CRV-FAM signals colocalizing with the F4/80-AF555 macrophage signals in the infected lungs. The peptides cleared primarily through the kidneys, with minor localizations seen in the spleen and liver. The results demonstrate the ability of the CRV peptide to home to macrophages in *S. aureus*-infected lungs.

Next, we tested the in vivo homing ability of the CRV when attached to the fusogenic NPs. Mice were intravenously injected, 24 h post infection, with DiI-loaded F-siIRF5 NPs coated with conjugated CRV peptide, and the in vivo biodistribution was tracked by immunofluorescence microscopy of organs harvested at 1 h and 24 h post treatment. The quantified DiI fluorescence signal showed strong accumulation in the lungs after 1 h and it was still detected 24 h post treatment (Fig. 4b and Supplementary Fig. 3 shows the representative images of the organs). Control experiments using the same NP construct but replacing the CRV-conjugated PEG with methoxylated PEG (F-DiI-mPEG) showed minimal accumulation in the lungs. The efficacy of lung homing was quantified by fluorescence-activated cell sorting (FACS), which showed that the CRV-conjugated fusogenic particles (F-DiI-CRV) homed to infected lungs with high selectivity (Supplementary Fig. 4**)**. Although the free-CRV peptide showed preferential clearance through the kidneys, the NPs showed no elevated fluorescence over background autofluorescence signals in the kidneys, and substantially increased clearance through the liver. These results were similar for both the targeted and the untargeted fusogenic NP constructs, in both healthy and infected animals. The clearance data are consistent with the size limitations displayed by the organs of mononuclear phagocytic system; NPs < 5.5 nm tend to be cleared by the kidneys and NPs show preferential clearance through the hepatobiliary system^40,41^. We note the CRV-conjugated NPs showed substantially enhanced uptake in the spleen of infected animals relative to controls, which is attributed to the splenic clearance and filtration of targeted macrophages that have phagocytosed senesced neutrophils and damaged cells from the infected lung.

We next studied the efficacy of CRV targeting and fusogenic uptake at the cellular level in the lungs using DiI-tagged NPs. The lungs of healthy mice intravenously injected with targeted fusogenic NPs (F-DiI-CRV) showed minimal recruitment of macrophages marked by F4/80-fluorescein isothiocyanate (FITC) and no visible F-DiI-CRV accumulation (Fig. 4c). Healthy lungs possess a baseline number of alveolar macrophages, with no significant recruitment of peripheral macrophages. However, the lungs of infected control mice (PBS injection at 24 h post infection) recruited a high number of macrophages, as expected of an inflammatory process (Fig. 4d). The group of infected mice injected (24 h post infection) with NF NPs (NF-DiI-CRV) showed minimal colocalization of macrophages with DiI from these NPs (Fig. 4e). In marked contrast, infected lungs of mice injected with the targeted fusogenic NPs (F-DiI-CRV) showed high accumulation of DiI signals, which were strongly colocalized with macrophage fluorescence signals (Fig. 4f). Taken together, the results show that the combination of CRV targeting and fusogenic uptake achieves strong homing to circulating macrophages recruited to infected lungs.

### Therapeutic efficacy of targeted fusogenic pSiNPs

**Fig. 5:**
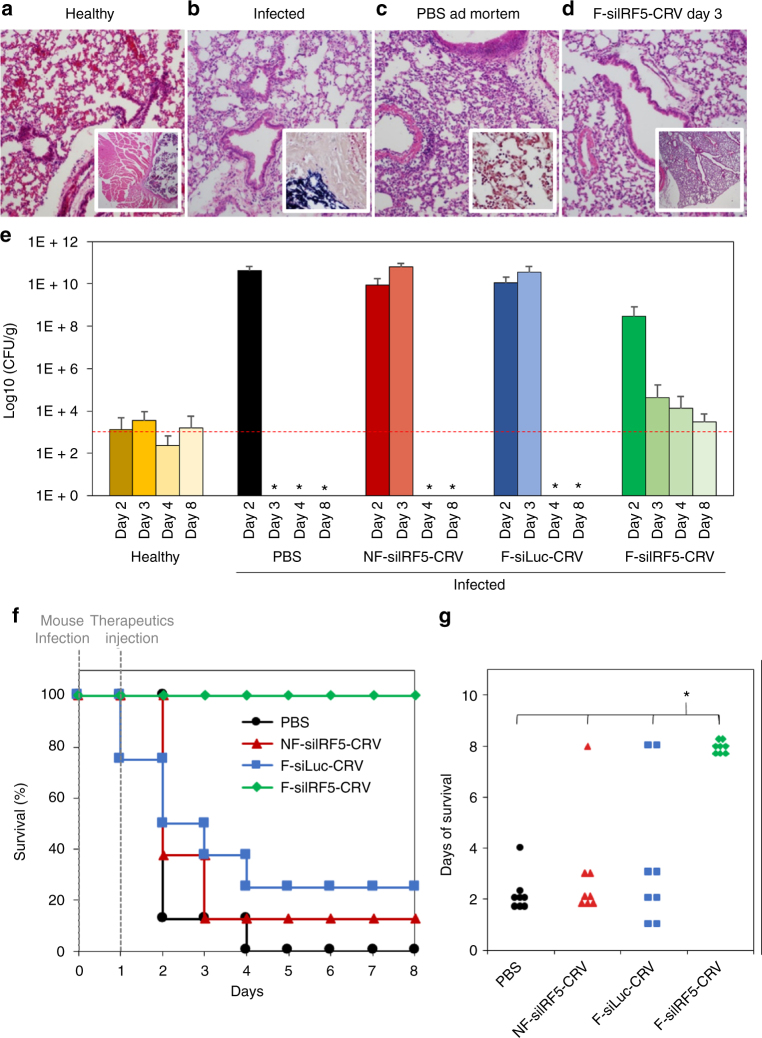
Macrophage-targeting fusogenic pSi nanoparticles loaded with si*Irf5* effectively treats infected mice. **a**–**d** H&E-stained sections of Balb/C mouse lungs subjected to histopathological analyses. **a** Lung of healthy mouse with no treatment (inset shows lower magnification of the same). **b** Lung of infected mouse with no treatment (inset shows large population of *S. aureus* in the perilaryngeal muscle). **c** Lung of infected mouse ad mortem 24 h post-PBS treatment (inset shows gram stain of *S. aureus* populations in the lung); **d** Lung of infected mouse treated with F-siIRF5-CRV nano-therapeutic (administered at 23.2 µmol/kg lipid, 24 µg/kg siRNA, 0.5 mg/kg pSi) at 3 days post treatment (inset shows lower magnification of the same). **e** Bacterial titer from lungs of healthy and infected mice injected with PBS, NF-siIRF5-CRV (non-fusogenic, targeted nanoparticle containing si*Irf5* therapeutic), F-siLuc-CRV (fusogenic, targeted nanoparticle containing siRNA against luciferase, as a negative control for si*Irf5*), and F-siIRF5-CRV (fusogenic, targeted nanoparticle containing si*Irf5* therapeutic). Animals were infected on day 0 and therapeutic or control injections were given on day 1. The dashed red line indicates the average count of colony-forming units (CFU) in healthy mouse lungs. Error bars indicate SD. Each bar represents *n* = 8 animals. *Ad mortem; no measurements due to death of all mice in the cohort. **f** Mouse survival post-infection (at day 0) and post-therapeutic injection (at day 1) of PBS, NF-siIRF5-CRV, F-siLuc-CRV, or F-siIRF5-CRV. Each group has *n* = 8 mice. **g** Average days of survival of mice from **f** post-infection and post-therapeutic injection. One-way ANOVA with Tukey’s HSD post hoc test (*p*-level < 0.05, *F* (3, 28) = 17, *p* = 1.77 × 10^−6^) revealed significant difference between F-siIRF5-CRV therapeutic and remaining three groups (PBS, NF-siIRF5-CRV, and F-siLuc-CRV). All animal experiments were performed independent of each other with different cohorts of mice

With the cellular fusion and in vivo homing capabilities established, we next evaluated the therapeutic efficacy of the fusogenic, siRNA-loaded, CRV-targeted nanosystem (F-siIRF5-CRV) against a lethal dose of *S. aureus* in the mouse pneumonia model. As discussed above, we chose to deliver siRNA against the *Irf5* gene of macrophages, in order to suppress inflammatory cytokine excretion of macrophages^13^ and enhance bacterial phagocytosis and tissue repair^10,15,16^. We initially studied bacterial colonization and titers in the lungs of mice in three treatment groups as follows: (i) *S. aureus*-infected mice with no treatment (lungs collected at fatality); (ii) infected mice treated with PBS (lungs collected at fatality within 24 h post treatment); and (iii) infected mice treated with F-siIRF5-CRV via tail vein injection (lungs collected 3 days post treatment). A section of a healthy lung is shown for comparison in Fig. 5a. The infected mice, when untreated (Fig. 5b) or treated with PBS only (Fig. 5c), displayed overt signs of neutrophilic pneumonia associated with bacteria. Moreover, *S. aureus* leakage into the perilaryngeal muscles was observed in these untreated controls (Fig. 5b, inset), and Gram-positive cocci were identified in the lungs of the PBS-injected group by Gram staining (Fig. 5c inset). Three days after injection of the F-siIRF5-CRV therapeutic, the lungs of the infected mice displayed an appearance similar to healthy lungs and no bacteria were detected in the Gram stains (Fig. 5d).

We also quantified the bacterial population in the various treatment groups by preparing lung homogenates and counting the number of bacterial colonies obtained (Fig. 5e). Healthy mice showed an average baseline count of ~ 10^3^ colony-forming unit (CFU)/g. Control groups of infected mice treated with PBS, with the NF targeted NP formulation containing si*Irf5* (NF-siIRF5-CRV), or with the fusogenic targeted NP containing siRNA against luciferase as a negative control for si*Irf5* (F-siLuc-CRV), were all observed to carry bacterial burdens of > 10^10^ CFU/g and all cohorts perished within 3 days of infection. By contrast, infected mice treated with the fusogenic targeted NP delivering the si*Irf5* therapeutic (F-siIRF5-CRV) demonstrated a notable decrease in titer starting 2 days post infection and titers reached the baseline count at 8 days post infection.

To confirm that the therapeutic effect resulted from *Irf5* knockdown, qRT-PCR was used to determine the relative in vivo knockdown efficiency in infected mice. Bronchoalveolar lavage (BAL) fluid collected from the lungs of infected mice was observed to have significantly lower expression of *Irf5* (17%) in mice injected with the CRV-conjugated fusogenic pSiNPs relative to the PBS, free si*Irf5*, CRV-conjugated NF-pSiNPs, and non-targeted fusogenic pSiNP controls (Supplementary Fig. 5a). However, the lung homogenates processed after removal of BAL fluid showed no significant difference in *Irf5* expression between all groups (Supplementary Fig. 5b). BAL fluid in healthy mice consists of 98% macrophages^42^, whereas infected mouse BAL fluid accumulates activated macrophages and neutrophils that are recruited during the initial stages of inflammation; by seven days post infection, the BAL fluid comprises 38% macrophages, 56% neutrophils, and 6% lymphocytes^42^. As the BAL fluid was collected 48 h post infection and 24 h post-therapeutic injection, the population is expected to be primarily macrophages. On the other hand, the lung homogenate is composed of epithelial, endothelial, and interstitial cells^43^, with a small population of macrophages. Thus, we conclude that the CRV-conjugated fusogenic particles successfully homed to the activated macrophages of the infected lungs in a selective manner and silenced *Irf5* gene expression.

In a separate experiment, mouse survival was dramatically improved with the F-siIRF5-CRV nano-therapeutic; 100% of the mice administered the formulation survived the lethal challenge and showed no apparent sequelae from the infection within a 4-day post-infection observation period (Fig. 5f). Mice administered the NF-siIRF5-CRV, and mice administered the F-siLuc-CRV, with siLuc as a sham siRNA, showed limited survival, with a significantly lower average number of survival days compared with the F-siIRF5-CRV (one-way analysis of variance (ANOVA), post hoc comparisons using Tukey’s honest significant difference (HSD) test, Fig. 5g). By comparison, the first-line antibiotic vancomycin gave only 30% survival rate in the same pneumonia model when intravenously administered at a 3 mg/kg (the published ED_50_ for vancomycin in mice is in the range 0.65–4 mg/kg^44,45^) single dose given one day post infection^46^.

## Discussion

A primary role of the inflammatory response during bacterial infection is to recruit additional macrophages whose function ultimately shifts to bacterial phagocytosis and tissue repair^10–15^. However, an excessive inflammatory reaction can become septic, generating multi-organ failure and fatality^2,3^. This study harnessed siRNA targeting the *Irf5* gene, a transcriptional regulator of the inflammatory M1 macrophage phenotype that is a key inductor of proinflammatory cytokines^11^. We hypothesized that effective silencing of this gene would suppress the inflammatory response and mitigate a lethal bacterial infection.

Silencing of *Irf5* has not previously been tested as a means to slow or eliminate bacterial infections. A serious bacterial infection, such as the lethal *S. aureus* model studied here, requires an overwhelming and effective immune response. Although *Irf5* is an attractive target, it was not clear that a strong response could be achieved using gene therapy alone, which can be notoriously inefficient in vivo^47^. Achieving high in vivo knockdown efficiency has been a challenge due to passive clearance in circulation and endocytic uptake that causes extracellular excretion or lysosomal degradation of the oligonucleotides^22,23^. In this work, we addressed these problems by incorporating three key features into the NP design: (i) a host pSiNP with high oligonucleotide loading efficiency and low systemic toxicity; (ii) a fusogenic lipid coating that effectively avoids endosomal uptake; and (iii) a targeting peptide that selectively homes the NPs to macrophages.

The first new aspect of the present approach was the NP host for the RNAi therapeutic. The carrier was based on porous silicon, a drug delivery vehicle that has shown good biocompatibility^48–50^, and an ability to load and protect sensitive therapeutics, such as proteins^51–53^ and oligonucleotides^35,54,55^. We used a self-sealing chemistry that trapped the oligonucleotide payload within the NP in a calcium silicate matrix^36^, loading a quantity at least 2 × greater than has been achieved with typical liposomal or related hybrid carriers (Supplementary Table 3). The calcium silicate pSiNPs follow the preparation protocol as introduced in Kang et al.^36^ In brief, large quantities of siRNA are loaded into pSi by precipitating a calcium silicate shell that simultaneously traps the payload. As the pSi matrix dissolves, the silicate product reacts with calcium (II) ion present in the CaCl_2_ solvent, and forms Ca_2_SiO_4_ at the NP surface. To load siRNA, the oligonucleotide is added to the solvent to trap the molecules during the Ca_2_SiO_4_ shell formation. Between the presented work and the referenced formulation, the pSi chemistry and properties were identical, with the exception of particle size (which was ~ 180 nm^36^). Thus, the pore volume in the F-pSi system is also expected to decrease by ~ 80% (1.36 ± 0.03 to 0.29 ± 0.04 cm^3^ g^−1^) upon shell formation. In contrast to the referenced work, the small pSiNP sizes (68.1 ± 5.8 nm; Supplementary Table 2) in the F-pSi system allows it to dissolve at an accelerated rate under physiological conditions (pH 7.4, 37 °C). Moreover, the higher mass loading is important in minimizing the injected dose and maximizing gene silencing in the cells. Also, unlike the cationic polymer or oligomer stabilizers usually employed to increase loading of negatively charged oligonucleotide payloads, Ca^2+^ is an endogenous species that is essential for cellular function.

Second, we introduced a liposomal coating that protected the NP from premature degradation until cellular fusion. The coating contained a specific composition of lipids that favored fusion with the cellular membrane over endocytosis (Fig. 2b–g). The fusogenic lipid coating was composed of pro-fusogenic lipids and moieties. 1,2-Dimyristoyl-sn-glycero-3-phosphocholine (DMPC) is the major constituent that acts as the structural backbone of the liposome, with a relatively low phase transition temperature (*T*_m_ = 24 °C). The low transition temperature gives the *L*_α_ liquid crystal phase at room temperature and at body temperature^56^. The *L*_α_ phase is the more fluidic, dynamic, and permeable structure that allows for a wide size range (100–400 nm) of extruded liposomal coatings and an easier fusion potential^57^. DOTAP is the cationic lipid essential for the electrostatic attraction toward the anionic plasma membrane^58,59^. Lastly, PEGylated lipid was also found to be imperative in fusion^31^; although the exact mechanistic role of PEG is not yet known, it is hypothesized that PEG binds water molecules to dehydrate the lipid head groups, which leads to structural asymmetry in the lipid alignment and drives double-leaflet to single-leaflet fusion as the energetically favorable route^60^, similar to how SNARE proteins anchor and pull vesicles into merging with plasma membranes endogenously; in fact, neuronal SNAREs have been observed to promote PEG-mediated fusion^61^. The fusion pathway peeled off the protective liposomal coating, which enhanced the rate of dissolution of the pSi carrier and release of the RNA payload after the bare NP was inserted into the cytosol, giving substantially greater knockdown of *Irf5* in vitro (Fig. 3a).

Third, we used an activated macrophage-specific targeting peptide, which provided strong and selective homing to macrophages in infected lungs. The effectiveness of the targeting peptide in enhancing gene silencing was not apparent in vitro due to the high efficacy of the fusogenic coatings (Fig. 3a). However, the targeting peptide was critical for effective homing to macrophages in infected lungs in vivo. The CRV-targeted NP constructs localized in infected and not healthy lungs (Fig. 4b, c, f), whereas NPs containing the sham targeting group showed little accumulation in either infected or healthy lungs (Fig. 4b). Furthermore, the CRV-conjugated NPs strongly colocalized with macrophages in infected lungs (Fig. 4f).

The siRNA therapeutic chosen for this study focused on enhancing the macrophage response to an infection by selectively inhibiting a gene associated with inflammatory M1 macrophages. Macrophages are essential components of the innate immune system that are responsible for defense against a wide range of pathogens. On a cellular level, the alveolar macrophages respond to a challenge of infectious particles by secreting cytokines, including TNF, IL-1β, IL-6, IL-8, IL-12, and IL-23 to recruit PMNs, which secrete additional cytokines. However, this prolonged excretion of inflammatory cytokines results in deleterious effects that drastically lower the chances of recovery^62^. In this study, we knock down *Irf5*, a key proinflammatory marker of M1 inflammatory macrophages^11^.

There are two leading hypotheses regarding the role of *Irf5* in macrophages. The first is that knockdown of *Irf5* in the M1 macrophage preserves its polarization, but eliminates proinflammatory factors^12^. It is known that knockdown of *Irf5* inhibits the expression of TNF and other inflammatory markers of M1 (e.g., *IL12A* and *IL23A*^11^), lowering recruitment of Th1/Th17 and impeding inflammation. In addition, a decrease in the TLR-mediated induction of proinflammatory cytokines (including IL-6) leads to further decrease in inflammation^63^. The result of eliminating the action of *Irf5* is an overall reduction in extended and damaging inflammation that occurs after initial leukocyte recruitment, preventing destruction of lung infrastructure and decreasing survival burden.

The second hypothesis is that *Irf5* knockdown causes M1 macrophages to repolarize to the M2 phenotype and become highly anti-inflammatory, yet strongly tissue-restorative^10^. In the M2 phenotype, IL-10 is upregulated and IL-12 is downregulated, the combination of which increases arginase-1 production to reduce nitrogen monoxide excretion and inflammation^10^. Moreover, the M2 macrophages are known to induce Th2 cytokine responses to mediate immune responses against extracellular bacteria and toxins^64^. Thus, in the re-polarization model, knockdown of IRF5 induces both anti-inflammatory tissue regeneration and immune effects to reduce infection burden and inhibit sepsis. Either model supports the hypothesis that knockdown of *Irf5* will improve healing of infections that induce an excessive inflammatory response. Indeed, in vivo silencing of *Irf5* has been shown to reduce inflammation and accelerate tissue regeneration in mouse models of myocardial infarct and skin wounds^10^. The improved healing response was attributed to attenuation of M1 macrophage polarization, which is typically the dominant macrophage phenotype in wounds shortly after injury. In these prior studies, the siRNA was delivered in a lipidoidal NP vehicle^65^.

Finally, it should be pointed out that the substantially improved survival afforded by the targeted gene nano-therapeutic developed in this work relative to a standardized dose of vancomycin represents a significant finding. Vancomycin is a first-line antibiotic, which is prescribed at high dosage and prolonged administration when used to treat *S. aureus* infections^5^. *S. aureus* strains have a history of evolving antibiotic-resistance genes, such that we are currently facing vancomycin-intermediate and -resistant strains that build strong peptidoglycan walls to bind and trap vancomycin, and inhibit its therapeutic action^2^. The *Irf5* knockdown approach used here is unlikely to be susceptible to development of resistance.

Taken together, the combination of high payload capacity, fusogenic uptake, and macrophage-specific targeting yielded an *Irf5*-silencing construct that rescued all mice tested from a lethal dose of *S. aureus*, and that gave the immune system time to clear the bacteria and return the lungs of the infected animals to their normal, healthy state within 7 days. The work reported here represents the first example of successful immunogene therapy against fatal deep-tissue infection. As the therapy focuses on changing the host macrophage response to suppress excessive inflammatory stimuli and enhance the antibacterial macrophage activity through *Irf5* silencing, rather than attacking a phenotypic characteristic of the pathogen, the approach should be applicable to a wide range of infections.

## Methods

### Materials

Highly boron-doped p-type silicon wafers (∼ 1 mΩ-cm resistivity, polished on the (100) face) were obtained from Virginia Semiconductor, Inc or Siltronix, Inc. Hydrofluoric acid (HF, 48% aqueous, ACS grade) was obtained from Fisher Scientific. Anhydrous calcium chloride was obtained from Spectrum Chemicals (Gardena, CA). Deionized (18 mΩ) water was used for all aqueous dilutions. For lipids, DMPC, 1,2-distearoyl-sn-glycero-3-phosphoethanolamine-N-[methoxy(PEG)-2000], 1,2-distearoyl-sn-glycero-3-phosphoethanolamine-N-[maleimide(PEG)-2000], and DOTAP were purchased from Avanti Polar Lipids (Alabaster, AL) and stored at − 4 °C. Fluorescent dyes Calcein (Sigma-Aldrich) and hydrophobic DiI (Life Technologies) were used and Lipofectamine® 2000 Transfection Reagent was obtained from Thermo Fisher Scientific. Custom siRNAs were purchased from Dharmacon (Lafayette, CO) and primers were purchased from IDT DNA (San Diego, CA). Macrophage-targeting peptide (CRV) was identified by Dr Erkki Ruoslahti’s group at Sanford Burnham Prebys Medical Discovery Center (SBPMDI, CA) and custom synthesized by CTC Scientific (Sunnyvale, CA). For in vitro studies, Raw 264.7 and J774a.1 cells were purchased from ATCC (Manassas, VA) within 6 months before all experiments. Dulbecco’s modified Eagle’s medium (DMEM) was purchased from GE Healthcare Life Sciences (HyClone, Pittsburg, PA), with supplemental fetal bovine serum (HyClone) and penicillin/streptomycin (HyClone). *S. aureus* subsp. aureus Rosenbach (ATCC® 25923™) was purchased from ATCC within 6 months before all experiments, and 6-week-old male Balb/C were purchased from Envigo (Placentia, CA).

### Animals

Six- to 8-week old mice (BALB/C, male) were purchased from Envigo (Indianapolis, IN). All animal experiments were performed independent of each other with different cohorts of mice. All animals for the in vivo studies were handled, anesthetized, and killed according to the Institutional Animal Care and Use Committee (IACUC) guidelines, and all experiments followed the approved protocol by the IACUC at SBPMDI.

### Preparation of pSiNPs

pSi samples were prepared by electrochemical etching of silicon wafers in an electrolyte consisting of 3:1 (v:v) of 48% aqueous HF:ethanol (caution: HF is highly toxic and proper care should be exerted to avoid contact with skin or lungs). A silicon working electrode with an exposed area of 8.6 cm^2^ was contacted on the back side with aluminum foil and mounted in a Teflon cell. The silicon wafer was then anodized in a two-electrode configuration with a platinum counter electrode, by applying an alternating current of square waveform, with lower current density of 50 mA/cm^2^ for 0.6 s and high current density of 400 mA/cm^2^ for 0.36 s repeated for 500 cycles. Then the porous layer was lifted off by etching at a constant current density of 3.7 mA/cm^2^ for 250 s in a 1:20 (v:v) of 48% aqueous HF:ethanol solution, to be sonicated in deionized water for 12 h into NPs. Fluorescent dye and siRNA payloads were loaded into the pSiNPs by pore sealing by calcium silicate formation; the calcium silicate sealing chemistry has demonstrated high efficiency in loading anionic payloads previously^36^. Calcein was dissolved in PBS at 100 mM. One hundred and fifty microliters of calcein was pipetted gently with 150 μl of pSiNP and 700 μl of 3 M calcium chloride under ultrasonication for 15 min. For siRNA loading, we used si*Irf5* (*Irf5*, sense 5′-dTdT-CUG CAG AGA AUA ACC CUG A-dTdT-3′ and antisense 5′-dTdT UCA GGG UUA UUC UCU GCA G dTdT-3′), and siLuc (luciferase, 5′-CUU ACG CUG AGU ACU UCG A-dTdT-3′ and antisense 5′-UCG AAG UAC UCA GCG UAA G dTdT-3′). siRNA was dissolved in RNAse-free water to 150 µM and loaded into pSi at the same volume ratio and process as calcein loading with only RNAse-free water used as solvent.

### Liposomal coating

Fusogenic coating (F) and NF coating were prepared from DMPC, DSPE-PEG, and DOTAP at the molar ratio of 76.2:3.8:20 and 96.2:3.8:0, respectively. The lipid films were prepared by evaporating the organic solvent, with 725.5 μg of DMPC, 151.6 μg of DSPE-PEG (methoxy or maleimide terminated), and 196.3 μg of DOTAP (F) or 916.0 μg of DMPC and 151.6 μg of DSPE-PEG (methoxy or maleimide terminated) (NF). The DiI-incorporated films were added with 26.3 μg of DiI (1.25 mg/ml in 100% ethanol). The films were then hydrated with payload-pSi solution and prepared by film hydration/extrusion; the pSi-hydrated lipid was heated to 40 °C with constant magnetic stirring for 10 min. Then the mixture was extruded through 200 nm polycarbonate membrane 20 times. CRV was conjugated to maleimide-terminated PEG by mixing 100 μl of 1 mg/ml CRV (in deionized water) in 1 mg/ml of the liposomal pSi (by lipid mass) overnight at 4 °C. Particles were washed three times at each step by centrifugation in Microcon-30 kDa Centrifugal Filter Unit (EMD Millipore) by spinning at 5000 × *g* at 25 °C. The loaded siRNA concentration was quantified by NanoDrop 2000 spectrophotometer (Thermo Fisher Scientific, ND-2000) after each step of particle formation by checking the ultraviolet absorption of the supernatant and pellet of each wash. NP size and zeta-potential were measured by DLS (Zetasizer ZS90, Malvern Instruments), and structural morphology were visualized by JEOL 1200 EX II TEM and FEI Tecnai Spirit G2 BioTWIN TEM. Samples were prepared by dropping 5 μl of the sample on the TEM grid, drying off excess solvent after 1 min, and dropping 5 μl of uranyl acetate for negative staining. Particle physiostability was observed by storing the formulations in PBS, refrigerated at 4 °C and measuring the hydrodynamic diameter every day for the first week, then every 7 days for a total of 4 weeks. The experiment was replicated three times and averaged.

### Macrophage-targeting peptide identification

Phage display screening was carried out as previously described^54^. Briefly, the macrophage cell line used here was J774A.1, a mouse monocyte/macrophage cell line isolated from ascites of female animals bearing reticulum cell sarcoma. A T7 phage library displaying 9-residue cyclic peptides (C*X*7C, two terminal Cysteine residues form a disulfide bond to render peptide cyclic; *X* being random amino acid) was used for screening. J774A.1 cells (2.5 × 10^5^) were first incubated with 5 × 10^10^ pfu (plaque-forming unit) inactivated phages displaying the sequence, RPARPAR, for 1 h at 4 °C. RPARPAR is a prototypic CendR (C-end Rule) peptide that will bind to a known receptor, neuropilin-1 (NRP1)^55^. Here, RPARPAR phages were inactivated for their infection ability by UV exposure before incubation with cells, and the pre-incubation of RPARPAR phage was to exclude all NRP1-binding peptide sequences. Next, 5 × 10^9^ pfu CX7C library was incubated with cells for another hour at 4 °C. Phage titering and subsequent rounds of enrichment were carried out according to our established protocol^54^. After three rounds of biopannings, the peptide sequences between two terminal Cysteine were determined using next generation sequencing performed by the DNA analysis core facility at SBPMDI.

### Peptide binding to cells

FAM-X-CRV (X being one copy of 6-aminohexanoic acid linker) and biotin-X-CRV were synthesized by Lifetein, LLC (Hillsborough, NJ). The control peptides used here included GGSGGSKG and ARA peptide^36^. Cells were incubated on rotator with indicated peptides at a final concentration of 10 µM in the solution of DMEM plus 1% bovine serum albumin. For competition study, biotin-X-CRV was added to a final concentration of 500 µM together with FAM-X-CRV (10 µM final). After 1 h at 4 °C, cells were washed three times with PBS followed by flow cytometry analysis.

### Affinity chromatography

The membrane proteins of Raw 264.7 cells were isolated using Mem-PER Plus membrane protein extraction kit (Thermo Fisher Scientific) according to the manufacturer’s instruction. Affinity chromatography was then performed with a protocol adapted from our previous report^54^. Briefly, biotin-X-CRV was first immobilized onto streptavidin coated magnetic beads (Thermo Fisher Scientific), then incubated with the membrane protein extracts of Raw 264.7 cells at 4 °C overnight. After two times of washing with PBS, beads were first incubated with 5′-GGSGGSKG-3′ peptide (a final concentration of 1 mM) at 4 °C for 3 h, to elute those nonspecifically bound proteins. Lastly, beads were incubated twice with FAM-CRV (a final concentration of 2 mM) at 4 °C (2 h each time), to elute those proteins specially bound to CRV. The elutes from 5′-GGSGGSKG-3′ (control peptide) and FAM-CRV were collected and subjected to mass spectrometry analysis by the mass spectrometry core facility at SBPMDI.

### Bacterial culture

All bacterial work was performed in an approved BSL-2 facility with a clean hood. *S. aureus* was cultured by incubating 50 μl of bacteria in 10 ml of the brain heart infusion broth (Fisher Scientific) for 16 h in a shaking incubator at 37 °C and shaking at 200 r.p.m. with the cap loose. The culture was re-introduced to the lag phase from the stationary phase by sub-culturing 10 μl of bacteria in fresh 5 ml of brain heart infusion broth for 2 h in a shaking incubator at 37 °C and shaking at 200 r.p.m. with the cap loose.

### Cell culture

Raw 264.7 and J774a.1 macrophage cell lines were cultured in DMEM supplemented with 10% FBS and 1% penicillin/streptomycin. All cells were incubated at 37 °C in 5% CO_2_.

Fusion of DiI-loaded or calcein-loaded nanoformulations were tested by seeding 6-well plates with 0.3 × 10^6^ cells on top of 22 mm round coverslips (BD Biocoat Collagen Coverslip, 22 mm), growing to 80% confluence overnight, and treating the cells with the NPs at 1 mg lipid dose. The formulations without CRV conjugations were incubated at 37℃ in 5% CO_2_ for 10 min, whereas the formulations with CRV conjugation were incubated for 5 min. Calcein-loaded particles were incubated for 10 min after cells were pre-treated with 500 nM of LysoTracker Red DND-99 (Thermo Fisher Scientific) for 1 h at 37 °C in 5% CO_2_. The NF particles generally demonstrate higher uptake (~ 3 ×) into cells relative to the fusogenic particles. For clearer visualization of the LysoTracker, the dose of F-pSi-Cal NF-pSi-Cal was decreased to 0.3 mg lipid dose. After incubation, the cells were washed in PBS three times to remove any particles that were not taken up. The cells were fixed in 1% paraformaldehyde (PFA, Santa Cruz Biotechnology) for 10 min, then washed with PBS three times. The coverslips were mounted on glass slides with ProLong® Diamond Antifade Mountant with DAPI (4',6-diamidino-2-phenylindole) (Life Technologies), dried and kept in the dark until examined by confocal microscopy (Zeiss LSM 710 NLO). PCC for colocalization was calculated using the Coloc2 plugin from ImageJ. At least ten representative images were analyzed to obtain the average coefficient.

For TEM of cells, particles were introduced to the Raw 264.7 and J774a.1 cells under the same conditions as above, and cells were fixed with glutaraldehyde overnight before being stained with osmium and uranyl acetate during embedding, and with lead on the TEM grids. The samples were viewed using a JEOL 1200 EX II TEM instrument.

### In vitro knockdown

In vitro knockdown efficiencies of the nanoformulations were quantified using two-step qRT-PCR (Roche LightCycler 96). Raw 264.7 cells were seeded on a six-well plate at 3 × 10^5^ cells per well and grown to 80% confluency overnight. The cells were incubated with the desired nanoformulations at 0.2 nmol of siRNA in 2 ml of media (100 nM siRNA). Forty-eight hours post incubation, the cell media was removed, and RNA was purified using the QIAshredder and RNeasy Mini Kit (Qiagen, Valencia, Ca). cDNA was transcribed from the purified RNA using the BIORAD iScript cDNA Synthesis Kit and heat-treated in the Eppendorf Vapo.protect Mastercycler thermal cycler. cDNA was mixed with *Irf5* primers, or the control hypoxanthine phosphoribosyltransferase (HPRT) primers (*Irf5* forward: 5′-AATACCCCACCACCTTTTG-3′; *Irf5* reverse: 5′-TTGAGATCCGGGTTTGAGAT-3′; HPRT forward: 5′-GTCAACGGGGGACATAAAAG-3′; HPRT reverse: 5′-CAACAATCAAGACATT-CTTTCCA-3′) and iQ SYBR Green Supermix according to the manufacturer’s instructions. RT-PCR analysis was performed in the BIORAD 96-well white Multiplate PCR Plates using the Roche LightCycler 96. The quantification was performed at *n* = 6 and in RNAse- and DNAse-free laminar flow hood dedicated to RNA work. Relative knockdown was statistically evaluated using one-way ANOVA with Tukey’s HSD post hoc analysis.

### Cell viability assay

Raw 264.7 and J774a.1 macrophages were cultured in six-well plates on top of 22 mm round coverslips (BD Biocoat) to 80% confluency from seeding density of 3 × 10^5^ cells/well. The cells were incubated for 1 h with NP formulations containing 0.5 mg and 1 mg total lipid mass. After incubation, cells were washed three times with PBS. Molecular Probes LIVE/DEAD Cell Viability/Cytotoxicity Kit (Thermo Fisher Scientific) uses calcein AM for live cell uptake (*λ*_ex_/*λ*_em_ = 494/517 nm) and EthD-1 for dead cell infiltration (*λ*_ex_/*λ*_em_ = 528/617 nm). The probes were treated and incubated with cells according to the manufacturer’s instructions. Briefly, probe stock was composed of 60 μl of 2 mM EthD-1 and 15 μl of 4 mM calcein AM in 30 ml fresh DMEM, and each cell well was incubated with 2 ml of the probe stock for 30 min at room temperature in the dark. The cells were then washed with PBS three times, fixed with 1% PFA, and mounted on glass slides. The slides were dried and imaged using confocal microscopy (Zeiss LSM 710 NLO) and the calcein AM/EthD-1 fluorescence signals were quantified using ImageJ at *n* = 20. The cell viability was statistically evaluated using one-way ANOVA. For fluorescence plate reader validation, the same experiment was conducted in a 96-well plate with seeding density of 6000 cells/well in 200 μl media and probe treatment. After 30 min room-temperature incubation in the dark, the fluorescence was read in a Gemini XPS spectrofluorometer (Molecular Devices) at *n* = 8 per group. The quantified viability was normalized to the PBS control treatment.

### Biosafety of fusogenic NPs

For in vivo biosafety validation, healthy Balb/C mice were intravenously injected with F-sIRF5-CRV at 23.2 µmol/kg lipid, corresponding to 69 µg/kg siRNA, and 0.3 mg/kg pSi in 100 μl PBS. After 24 h circulation, the mice were killed under deep isofluorane anesthesia (no response to toe pinch) by cardiac perfusion, and the brain, heart, lungs, liver, kidneys, and spleen were collected. Organs were fixed immediately in 4% PFA and sent to the University of California, San Diego (UCSD)’s histology core to be paraffinized and sectioned for H&E staining. The stained slides were histopathologically evaluated by Dr Kent Osborn (Associate Director, Animal Care Program, UCSD).

### In vivo infection model

After 16 h incubation in brain heart infusion broth, 10 μl *S. aureus* was sub-cultured in 5 ml of fresh broth for 2 h to reach growth phase. The optical density at 600 nm was measured using a cuvette spectrometer with the broth set as the blank. Five milliliters of bacterial culture at OD_600_ ≈ 0.32 was centrifuged, the bacteria were washed by centrifugation in PBS three times, and re-suspended in 200 μl of PBS for inoculation. The *S. aureus* pneumonia animal model was established in 6- to 8-week-old male Balb/C mice by intratracheal catheter injection of ~ 1 × 10^7^ CFU of bacteria in 10 μl of PBS. All treatment injections were performed 24 h after inoculation of the bacteria.

### Biodistribution of CRV

Three healthy and infected (24 h post infection) mice were intravenously injected with CRV tagged with 5’6-FAM dye in 100 μl PBS at a concentration of 1 mg/ml. Organs were harvested after 1 h circulation and fixed in 4% PFA. Organs were sent to the UCSD histology core to be paraffinized, sectioned, and stained with DAPI nuclei stain and F4/80-AF555 macrophage marker for immunofluorescence analysis under the Zeiss LSM 710 NLO confocal microscope with single photon laser (for excitation of DiI, calcein, LysoTracker Red) and Mai-Tai Laser HB (690-1020 nm) (for two-photon excitation of DAPI).

### NP biodistribution

Eight-week-old male Balb/C mice were intratracheally infected as described. Twenty-four hours post infection, infected and healthy mice were intravenously injected with si*Irf5*-loaded fusogenic pSiNPs with or without CRV conjugation, at 23.2 µmol/kg lipid, corresponding to ~ 69 µg/kg siRNA, and 0.3 mg/kg pSi 100 μl in PBS. The DiI-loaded particle localization was visualized using the IVIS 200 (Perkin Elmer) with 0.12 s exposure time on the DsRed excitation and emission filters. Both healthy and infected animals were killed and collected for organs 24 h post infection, with additional 1 h post-treatment analyses for infected animals injected with the fusogenic NP formulations. ImageJ was used to quantify the fluorescence of each organ, and averaged over the three mice per group. Infected lung homing was further validated using FACS. Twenty-four hours post infection, mice were intravenously injected with calcein-loaded NF particles with CRV, fusogenic particles without CRV, and fusogenic particles with CRV at 23.2 µmol/kg lipid, corresponding to 69 µg/kg siRNA, and 0.3 mg/kg pSi in 100 μl PBS. One hour post injection, the mice were killed by cardiac perfusion with PBS. The collected lungs were homogenized and the homogenates were processed with the LSR Fortessa FACS instrument, and analyzed using the FlowJo software (FlowJo, LLC). For immunofluoresce microscopy of infected lungs, mice were intravenously injected 24 h post infection with DiI-loaded formulations of NF and fusogenic particles (without siRNA) conjugated with CRV, and were killed for lung collection and fixation in 4% PFA at 24 h post injection. The fixed lungs were paraffinized and sectioned, and stained with FITC-labelled F4/80 macrophage marker. The sections were observed under Zeiss LSM 710 NLO confocal microscope for DiI and FITC localizations.

### In vivo therapeutic efficacy of fusogenic NPs

Eight-week-old male Balb/C mice were intratracheally infected as described. Twenty-four hours post infection, infected mice were intravenously injected with 100 μl of PBS or si*Irf5*-loaded fusogenic pSiNPs conjugated with CRV at 23.2 µmol/kg lipid, corresponding to 69 µg/kg siRNA, and 0.3 mg/kg pSi in 100 μl PBS. PBS-injected mouse was collected for the lungs ad mortem (24 h post injection), whereas nanoformulation-injected mouse was killed and collected for the lungs 3 days post injection. In addition, lungs from healthy mouse and infected mouse with no injection, at 24 h post infection, were collected. All organs were immediately fixed in 4% PFA to be sent to the UCSD histology core for paraffinization, sectioning, and H&E staining. The stained sections were histopathologically evaluated by Dr Kent Osborn (Associate Director, Animal Care Program, UCSD).

To confirm clearance of bacteria from lungs, the number of CFUs of *S. aureus* was determined from titering of lung homogenates. Twenty-four hours post infection, mice were intravenously injected with 100 μl of PBS, NF particles with si*Irf5* and CRV conjugation, fusogenic particles with sham siRNA (siLuc, luciferase encoding siRNA) conjugated with CRV, or fusogenic particles with si*Irf5* and CRV conjugation at 23.2 µmol/kg lipid, corresponding to 69 µg/kg siRNA, and 0.3 mg/kg pSi in 100 μl PBS. Healthy mice with no injection were also observed. At days 2, 3, 4, and 8 post infection, mice were killed for lung collection. Due to factors that may affect the bacteria count on the day of the therapeutics injection, day 1 (i.e., variations in before vs. after injection, the number of hours post injection, etc.), we began the count at day 2. In cases where mice succumbed to infection before the time points, the lungs were collected ad mortem. The lungs were weighed, gently washed in PBS, and then homogenized. The homogenates were serially diluted to a dilution factor of 10^7^, plated on agar-coated petri dishes, and incubated at 37 °C overnight. The *S. aureus* colonies were counted *n* = 4 for each dilution factor and divided by the lung mass. The average CFU/g was quantified using counts from four plates at equivalent dilution factors from two mice (*n* = 4 plates × 2 mice = 8).

Finally, a survival challenge was performed with infected mice, who were intravenously injected 24 h post infection with the treatment compounds. Each group had eight mice, which were blindly observed daily for survival. Moribund mice that showed signs of expiring within 5 h were killed according to the IACUC guidelines. The resulting data were statistically evaluated using single-way ANOVA and post hoc comparisons using Tukey’s HSD test at *p* < 0.05.

### In vivo *Irf5* knockdown efficiency

Eight-week-old male Balb/C mice were intratracheally infected as described above. Twenty-four hours post infection, infected mice were intravenously injected with 100 μl of PBS or si*Irf5*-loaded fusogenic and NF-pSiNPs with or without CRV at 23.2 µmol/kg lipid, corresponding to 69 µg/kg siRNA, and 0.3 mg/kg pSi in 100 μl PBS. Twenty-four hours post injection and circulation, mice were killed for BAL. BAL was performed by intratracheal instillation of a mouse catheter, with a suture tied around the trachea to prevent leakage. One milliliter of PBS was injected into the lungs through the catheter and aspirated back out. The process was repeated three times to collect up to 2.5 ml of the BAL fluid. Lungs were also collected after completion of the BAL procedure.

Cells from the BAL fluid were collected by centrifugation at 350 × *g* for 10 min at room temperature. The supernatant was removed and the cell pellets were washed with PBS once by centrifugation at 350 × *g* for 10 min at room temperature. Before qRT-PCR processing, the cell pellets were kept dry at − 80 °C. Collected lungs were weighed and homogenized, 30 mg of the homogenates were isolated for qRT-PCR processing, and stored at − 80 °C.

The in vivo knockdown of *Irf5* was quantified using two-step qRT-PCR (Roche LightCycler 96). The cell pellets or the lung homogenates were lysed and RNA was purified using the QIAshredder and RNeasy Mini Kit (Qiagen). cDNA was transcribed from the purified RNA using the BIORAD iScript cDNA Synthesis Kit and heat-treated in the Eppendorf Vapo.protect Mastercycler thermal cycler. cDNA was mixed with *Irf5* primers, or the control HPRT primers (*Irf5* forward: 5′-AATACCCCACCACCTTTTGA-3′; *Irf5* reverse: 5′-TTGAGATCCGGGTTTGAGAT-3′; HPRT forward: 5′-GTCAACGGGGGACATAAAAG-3′; HPRT reverse: 5′-CAACAATCAAGACATT-CTTTCCA-3′) and iQ SYBR Green Supermix according to the manufacturer’s instructions. qRT-PCR analysis was performed in the BIORAD 96-well white Multiplate PCR Plates using the Roche LightCycler 96. The quantification was performed at *n* = 6 and in RNAse- and DNAse-free laminar flow hood dedicated to RNA work. Relative knockdown was statistically evaluated using one-way ANOVA with Tukey’s HSD post hoc analysis.

### Data availability

The data that support the findings of this study are available from the corresponding author upon request.

## Electronic supplementary material

Supplementary Information
