# Supplementary material for: Immunogene therapy with fusogenic nanoparticles modulates macrophage response to Staphylococcus aureus
Source: Nat Commun. 2018 May 17;9:1969. doi: 10.1038/s41467-018-04390-7 (PMC5958120; doi:10.1038/s41467-018-04390-7)
Supplement: Supplementary file 1 — Supplementary Information [file 41467_2018_4390_MOESM1_ESM.pdf]

Supplementary Information

Immunogene therapy with fusogenic nanoparticles modulates macrophage response to *Staphylococcus aureus*

Byungji Kim[†,1], Hong-Bo Pang[†,2], Jinyoung Kang[3], Ji-Ho Park[4], Erkki Ruoslahti[2,5], and Michael J. Sailor*[1,3,6]

[1] Materials Science and Engineering Program, University of California, San Diego, 9500 Gilman Dr., La Jolla, CA 92093, USA

[2] Cancer Research Center, Sanford Burnham Prebys Medical Discovery Institute, La Jolla, California 92037, USA

[3] Department of Nanoengineering, University of California, San Diego, 9500 Gilman Dr., La Jolla, CA 92093, USA

[4] Department of Bio and Brain Engineering, Korea Advanced Institute of Science and Technology (KAIST), Daejeon 34141, Republic of Korea

[5] Center for Nanomedicine and Department of Cell, Molecular and Developmental Biology, University of California, Santa Barbara, Santa Barbara, California 93106-9610, USA

[6] Department of Chemistry and Biochemistry, University of California, San Diego, 9500 Gilman Dr., La Jolla, CA 92093, USA

*Corresponding author. E-mail: msailor@ucsd.edu

[†]Equal contribution

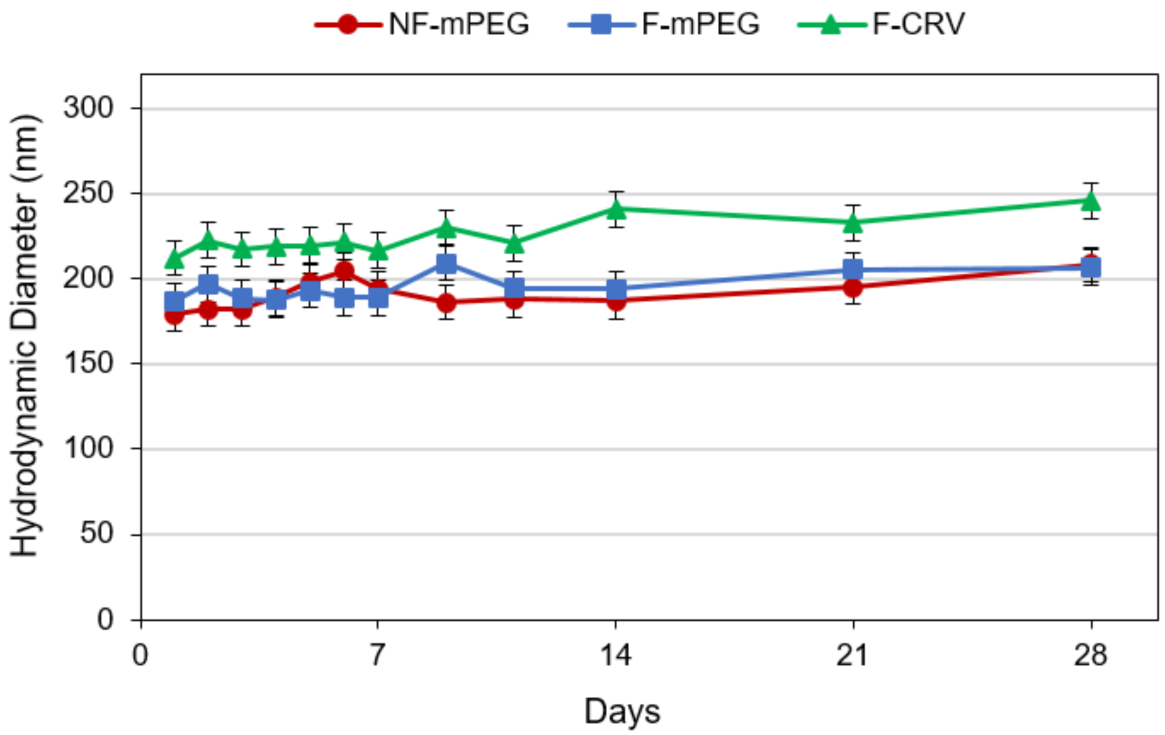

Supplementary Figure 1. Nanoparticles demonstrate physical stability for up to 28 days in physiological salt solution. 28-day observation of average hydrodynamic diameter of fusogenic (F-mPEG) and non-fusogenic (NF-mPEG) porous silicon (pSi) nanoparticles, and CRV-conjugated fusogenic porous silicon nanoparticles (F-CRV) in aqueous phosphate-buffered saline (PBS) solution, measured by DLS. Bars indicate standard deviation, n=3.

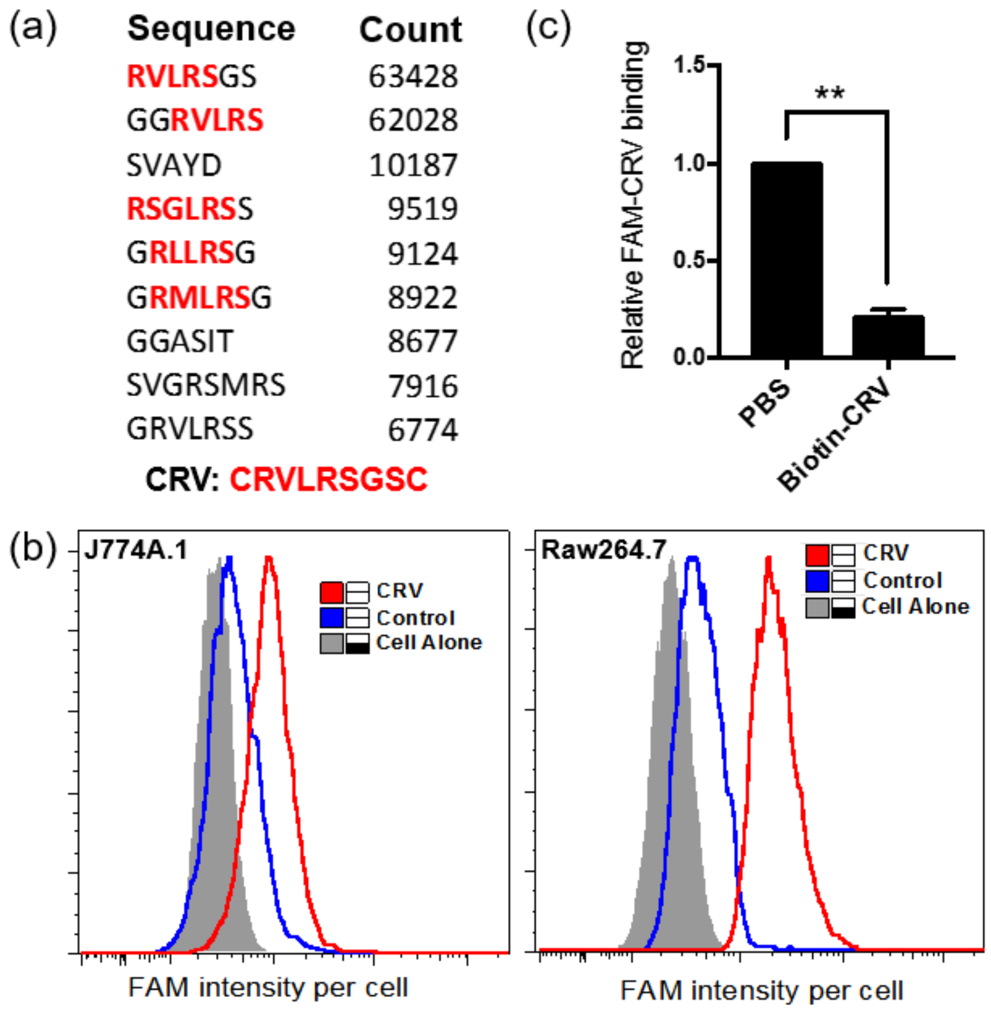

Supplementary Figure 2. Identification of CRV peptide and *in vitro* characterization of macrophage binding. (a) Phage display using CX_7C library was performed on J774A.1 cells as described in Materials and Methods. The amino acid sequences between two Cysteine residues of the most abundant phages enriched after three rounds of biopannings, and their frequency, are shown here. A consensus motif, RVLRS, is highlighted here in red. (b) FAM-CRV, or a FAM labeled control peptide (ARA for J774A.1 cells; GGSGGSKG for Raw264.7 cells) was mixed with indicated cells for binding as described in Materials and Methods. At least three independent experiments were carried out, and the representative results are shown here. (c) FAM-CRV was mixed with PBS or biotin-CRV for competition of binding to J774A.1 cells as described in Materials and Methods. Error bars, SEM. **P<0.01 (Student's t-test).

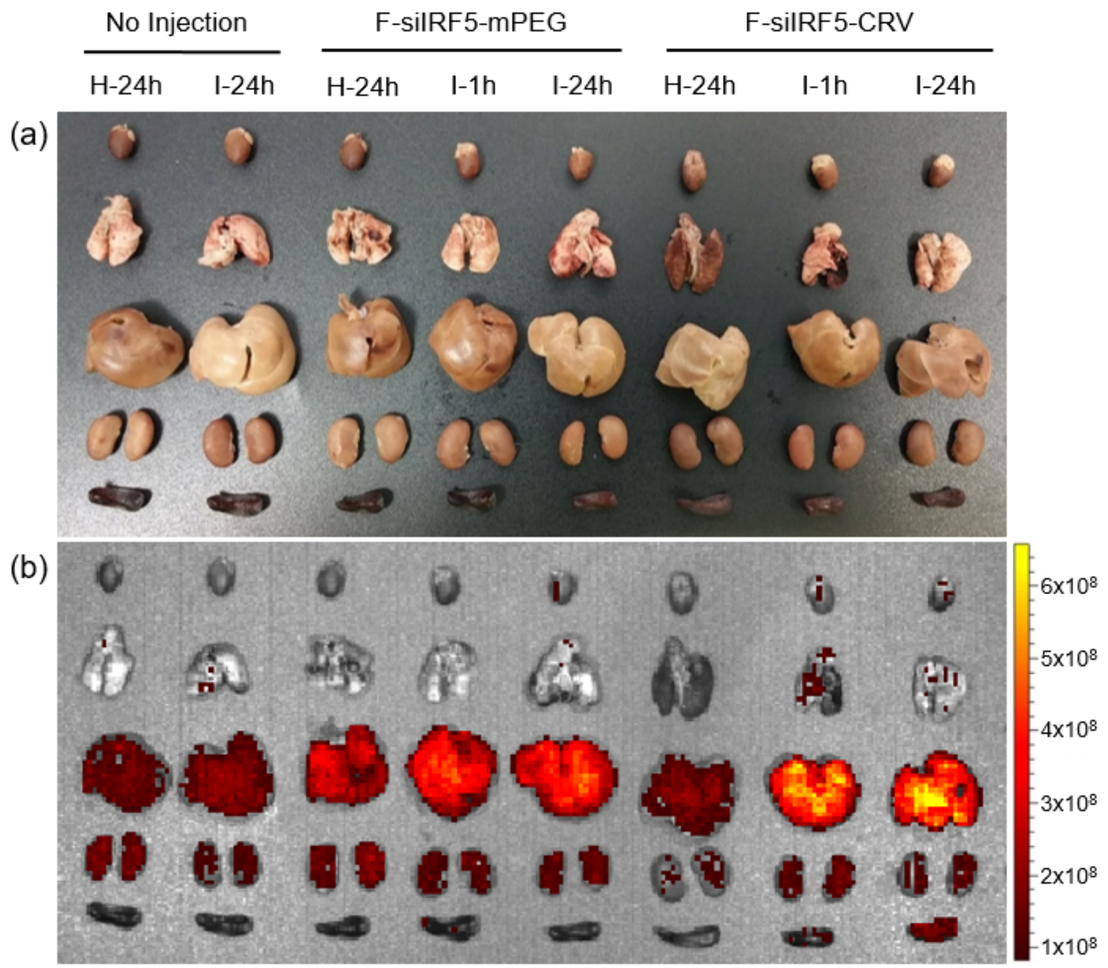

Supplementary Figure 3. CRV-conjugated fusogenic particles show homing to infected lungs within 1h of intravenous injection. Representative images of biodistribution of DiI-tagged fusogenic nanoparticles in healthy and infected Balb/C mice. (a) Photograph of harvested organs (top to bottom: heart, lungs, liver, kidneys, and spleen); (b) IVIS 200 fluorescence image of DiI-labeled fusogenic nanoparticle without CRV conjugation (F-siIRF5-mPEG) and fusogenic nanoparticle with CRV conjugation (F-siIRF5-CRV). 'H-24' indicates healthy Balb/C organs harvested 24h post-treatment, 'I-1' indicates organs harvested from infested mice 1h post-treatment, and 'I-24' indicates infected Balb/C organs 24h post-treatment. Data are representative of n=3. The images were quantified using ImageJ software, given in **Fig. 4b**.

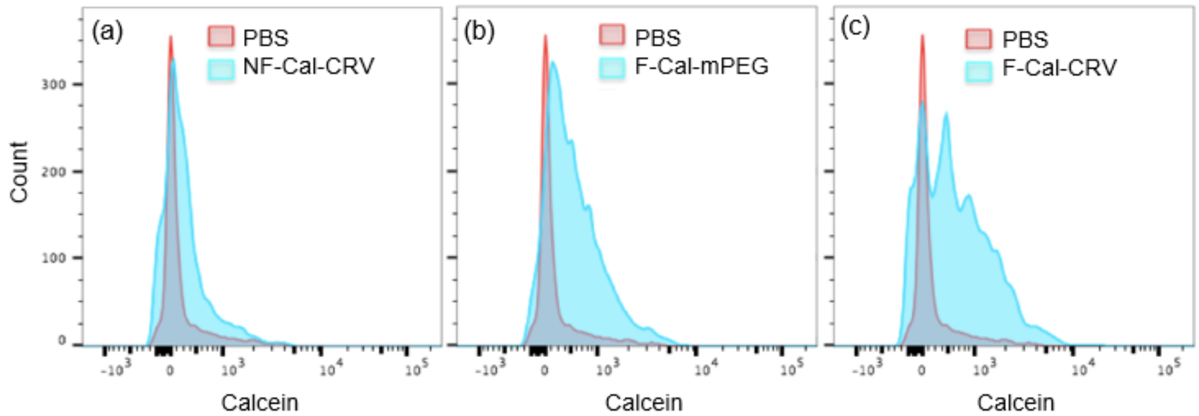

Supplementary Figure 4. CRV-conjugation enhances selective homing to infected lungs. FACS analyses of calcein accumulation in homogenized Staph. aureus-infected Balb/C lungs. (a) PBS vs. non-fusogenic particles loaded with calcein (Cal) and conjugated to targeting peptide (CRV); (b) PBS vs. fusogenic particles loaded with calcein without targeting peptide; (c) PBS vs. fusogenic particles loaded with calcein and conjugated to targeting peptide. These data validate the efficacy of CRV-conjugation. Infected Balb/C were intravenously injected with the indicated preparations. Lungs were harvested 1h post-injection and homogenized. The homogenates were analyzed by FACS for cellular accumulation of calcein-loaded particles. NF-Cal-CRV showed no visible difference in calcein signal compared to PBS-injected mice. Non-targeted F-Cal-mPEG showed a slight shift in the calcein signal toward higher values, but targeted F-Cal-CRV demonstrated a substantial peak shift toward a higher degree of fluorescence. The data demonstrate that CRV-conjugation to fusogenic particles enables homing to infected lungs.

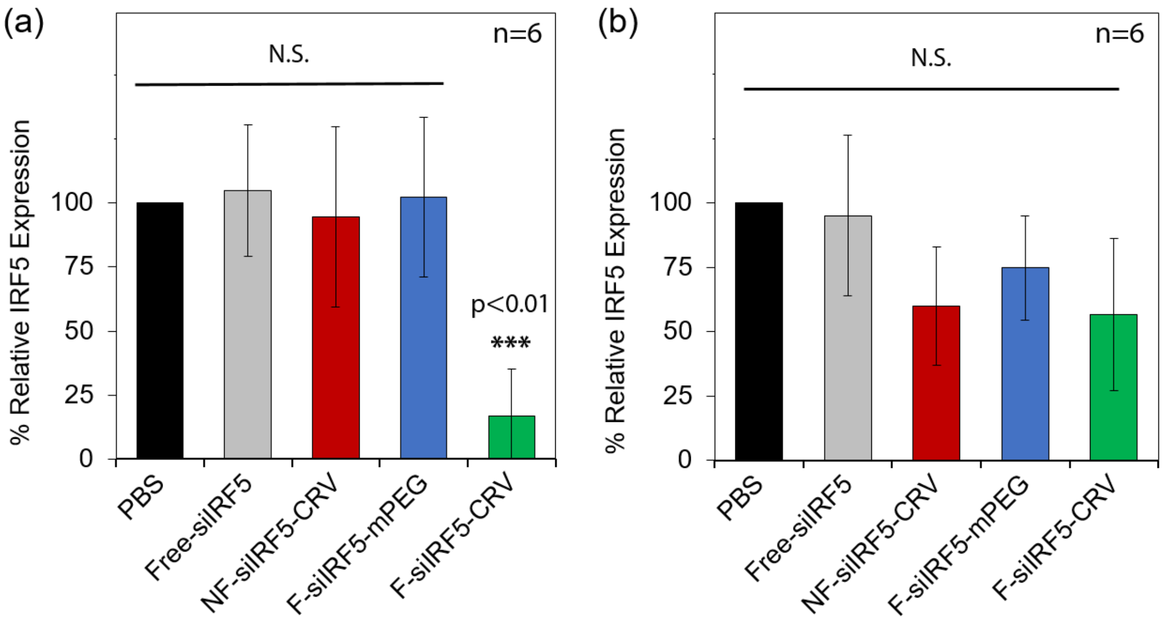

Supplementary Figure 5. CRV-conjugated fusogenic pSi nanoparticles demonstrate effective knockdown of IRF5 in the BAL fluid of infected lungs *in vivo*. (a) *in vivo* siRNA knockdown efficiency (via qRT-PCR) in BAL fluid collected from *Staph. aureus* infected Balb/C mice injected with formulations for 24h. Error bars indicate standard deviation (n=6). *** indicates significant difference (One-way ANOVA with Tukey's HSD post hoc test, p level <0.05, F (5, 30) = 26.5, p = 5.9×10^{-14}). **(b)** *in vivo* siRNA knockdown efficiency (via qRT-PCR) in lung homogenates from *Staph. aureus* infected Balb/C mice injected with nanoparticles for 24h. Error bars indicate standard deviation (n=6). N.S. indicates no significant difference.

Supplementary Table 1. Lipid composition of fusogenic and non-fusogenic liposomal coatings expressed in molar ratio, where DMPC is 1,2-dimyristoyl-sn-glycero-3-phosphocholine, DSPE-PEG is 1,2-distearoyl-sn-glycero-3-phosphoethanolamine-N-[methoxy(polyethylene glycol)-2000] (DSPE-mPEG), or 1,2-distearoyl-sn-glycero-3-phosphoethanolamine-N-[maleimide(polyethylene glycol)-2000] (DSPE-PEG-maleimide), and DOTAP is 1,2-dioleoyl-3-trimethylammonium-propane.

	**Lipid Composition (Molar Ratio)**		
	**DMPC**	**DSPE-PEG**	**DOTAP**
**Fusogenic**	76.2	3.8	20
**Non-fusogenic**	96.2	3.8	0
**Fusion mechanism**	• Major structural component<br>• Low phase transition temperature allows for liquid crystal phase (essential phase for fusion) in physiological conditions	• For dispersion and stealth<br>• Hypothesized to act as a bridge linker akin to SNARE<br>• PEG-methoxy used for non-conjugated<br>• PEG-maleimide used for targeting peptide conjugation	• Cationic lipid for fusion and attraction to plasma membrane

Supplementary Table 2. Table of particle size and zeta-potential measured by DLS (n=3). F-mPEG indicates fusogenic liposome-coated pSi nanoparticles without CRV conjugation; NF-mPEG indicates non-fusogenic liposome-coated pSi nanoparticles without CRV conjugation; F-CRV indicates fusogenic liposome-coated pSi nanoparticles with CRV conjugation; and NF-CRV indicates non-fusogenic liposome-coated pSi nanoparticles with CRV conjugation.

	Average Hydrodynamic Diameter (nm)	Zeta-Potential (mV)
Core pSiNPs	68.1 ± 5.8	-21.3 ± 1.0
Fusogenic (F-mPEG)	187.4 ± 5.2	9.8 ± 0.4
Non-fusogenic (NF-mPEG)	190.8 ± 4.7	-9.1 ± 1.8
Targeted Fusogenic (F-CRV)	225.0 ± 10.2	-3.4 ± 2.3
Targeted Non-fusogenic (NF-CRV)	229.2 ± 7.8	-10.8 ± 0.8

Supplementary Table 3. siRNA loading efficiency by wt.% comparison between Fusogenic pSiNPs and conventional platforms compiled based on literature published from 2008-2016.

Particle	Size	siRNA Loading (wt. %)
Fusogenic liposome-coated pSiNP	190 nm	20-25%
Lipid-based Nanoparticles	50-200 nm	1-14%†
Mesoporous Silica-Polymer Hybrid NPs	60-200 nm	1-10%‡

† Comparable 200 nm particles had the lowest loading amounts (< 5 wt. %)[1-11]
‡ Comparable 200 nm particles had the lowest loading amounts (< 5 wt. %)[12-16]

Supplementary References

1 Auguste, D. T. *et al.* Triggered release of siRNA from poly(ethylene glycol)-protected, pH-dependent liposomes. *J. Control. Release* **130**, 266-274, doi:10.1016/j.jconrel.2008.06.004 (2008).

2 Basha, G. *et al.* Influence of cationic lipid composition on gene silencing properties of lipid nanoparticle formulations of siRNA in antigen-presenting cells. *Mol. Ther.* **19**, 2186-2200, doi:10.1038/mt.2011.190 (2011).

3 Leuschner, F. *et al.* Therapeutic siRNA silencing in inflammatory monocytes in mice. *Nat. Biotechnol.* **29**, 1005-1010, doi:10.1038/nbt.1989 (2011).

4 Sahay, G. *et al.* Efficiency of siRNA delivery by lipid nanoparticles is limited by endocytic recycling. *Nat. Biotechnol.* **31**, 653-658, doi:10.1038/nbt.2614 (2013).

5 Gilleron, J. *et al.* Image-based analysis of lipid nanoparticle-mediated siRNA delivery, intracellular trafficking and endosomal escape. *Nat. Biotechnol.* **31**, 638-646, doi:10.1038/nbt.2612 (2013).

6 Akinc, A. *et al.* Targeted delivery of RNAi therapeutics with endogenous and exogenous ligand-based mechanisms. *Mol. Ther.* **18**, 1357-1364, doi:10.1038/mt.2010.85 (2010).

7 Akinc, A. *et al.* Development of lipidoid-siRNA formulations for systemic delivery to the liver. *Mol. Ther.* **17**, 872-879, doi:10.1038/mt.2009.36 (2009).

8 Xu, C.-f. & Wang, J. Delivery systems for siRNA drug development in cancer therapy. *Asian J. Pharmacol.* **10**, 1-12, doi:http://dx.doi.org/10.1016/j.ajps.2014.08.011 (2015).

9 Leung, A. K. *et al.* Lipid Nanoparticles Containing siRNA Synthesized by Microfluidic Mixing Exhibit an Electron-Dense Nanostructured Core. *J. Phys. Chem. C* **116**, 18440-18450, doi:10.1021/jp303267y (2012).

10 Zheng, Y. *et al.* A novel gemini-like cationic lipid for the efficient delivery of siRNA. *New J. Chem.* **38**, 4952-4962, doi:10.1039/C4NJ00531G (2014).

11 Hasan, W. *et al.* Delivery of Multiple siRNAs Using Lipid-Coated PLGA Nanoparticles for Treatment of Prostate Cancer. *Nano Lett* **12**, 287-292, doi:Doi 10.1021/Nl2035354 (2012).

12 Xia, T. A. *et al.* Polyethyleneimine Coating Enhances the Cellular Uptake of Mesoporous Silica Nanoparticles and Allows Safe Delivery of siRNA and DNA Constructs. *ACS Nano* **3**, 3273-3286, doi:10.1021/nn900918w (2009).

13 Bhattarai, S. R. *et al.* Enhanced Gene and siRNA Delivery by Polycation-Modified Mesoporous Silica Nanoparticles Loaded with Chloroquine. *Pharm. Res.* **27**, 2556-2568, doi:10.1007/s11095-010-0245-0 (2010).

14 Moller, K. *et al.* Highly efficient siRNA delivery from core-shell mesoporous silica nanoparticles with multifunctional polymer caps. *Nanoscale* **8**, 4007-4019, doi:10.1039/c5nr06246b (2016).

15 Shen, J. *et al.* Cyclodextrin and polyethylenimine functionalized mesoporous silica nanoparticles for delivery of siRNA cancer therapeutics. *Theranostics* **4**, 487-497, doi:10.7150/thno.8263 (2014).

16 Ashley, C. E. *et al.* Delivery of small interfering RNA by peptide-targeted mesoporous silica nanoparticle-supported lipid bilayers. *ACS Nano* **6**, 2174-2188, doi:10.1021/nn204102q (2012).
